# Amylase and Xylanase from Edible Fungus *Neurospora intermedia*: Production and Characterization

**DOI:** 10.3390/molecules24040721

**Published:** 2019-02-17

**Authors:** Zohre Shahryari, Mohammad H. Fazaelipoor, Younes Ghasemi, Patrik R. Lennartsson, Mohammad J. Taherzadeh

**Affiliations:** 1 Swedish Centre for Resource Recovery, University of Borås, SE-50190 Borås, Sweden; patrik.lennartsson@hb.se (P.R.L.); mohammad.taherzadeh@hb.se (M.J.T.); 2 Department of Chemical Engineering, Faculty of Engineering, Shahid Bahonar University of Kerman, Kerman 7618868366, Iran; fazaelipoor@yazd.ac.ir; 3 Department of Chemical and Polymer Engineering, Faculty of Engineering, Yazd University, Yazd 8915818411, Iran; 4 Pharmaceutical Sciences Research Center, Shiraz University of Medical Sciences, Shiraz 71345, Iran; ghasemiy@sums.ac.ir; 5 Department of Pharmaceutical Biotechnology, School of Pharmacy, Shiraz University of Medical Sciences, Shiraz 7146864685, Iran

**Keywords:** amylase, xylanase, *Neurospora intermedia*, submerged fermentation, wheat-based biorefinery

## Abstract

Integrated enzyme production in the biorefinery can significantly reduce the cost of the entire process. The purpose of the present study is to evaluate the production of two hydrolyzing enzymes (amylase and xylanase) by an edible fungus used in the biorefinery, *Neurospora intermedia*. The enzyme production was explored through submerged fermentation of synthetic media and a wheat-based waste stream (thin stillage and wheat bran). The influence of a nitrogen source on *N. intermedia* was investigated and a combination of NaNO_3_ and yeast extract has been identified as the best nitrogen source for extracellular enzyme production. *N. intermedia* enzymes showed maximum activity at 65 °C and pH around 5. Under these conditions, the maximum velocity of amylase and xylanase for starch and xylan hydrolysis was found to be 3.25 U mL^−1^ and 14.77 U mL^−1^, respectively. Cultivation of *N. intermedia* in thin stillage and wheat bran medium resulted in relatively high amylase (8.86 ± 0.41 U mL^−1^, 4.68 ± 0.23) and xylanase (5.48 ± 0.21, 2.58 ± 0.07 U mL^−1^) production, respectively, which makes this fungus promising for enzyme production through a wheat-based biorefinery.

## 1. Introduction

Amylases and xylanases are hydrolytic enzymes that contribute in saccharification processes to assist the hydrolysis of starch and xylan, respectively. Starch is the most common carbohydrate in the human and animal diet [1]. Xylan is also the second most abundant natural polysaccharide and the major structural component of plant cell walls [2]. Considering the vast application of starch and xylan in food, feed, textile, pulp and paper, brewing, juice, and wine industries, amylases and xylanases have received a great deal of attention, especially in these industries [1,3].

These enzymes can be obtained from animals, plants, and microorganisms. However, enzymes produced by microorganisms are more preferable to plant and animal based ones owing to their high yields and reliability, higher stability, possibility of product modification and optimization, economic feasibility, regular supply because of the absence of seasonal fluctuations, a rapid growth of microbes on low cost media, ease of cultivation in large fermenters, and greater catalytic activity [4]. In addition, enzyme production using microorganisms creates the possibility of on-site enzyme production by utilization of residual streams resulting in the biorefinery, which reduces the cost of the process.

A number of microbial sources including bacteria and fungi have been reported for amylase and xylanase production under the fermentation process [5,6]. Nevertheless, fungi-derived enzymes have obtained considerable attention due to the ease of cultivation, high production yields, and simultaneous biomass production [7]. Among fungi, specific interest has been expressed in a sort of edible fungi that can naturally synthesize and secrete enzymes. The application of edible fungi in enzyme production enhances the chance of the enzymes and biomass being free from contamination from mycotoxins (toxins of fungal origin [8]).

The ascomycete filamentous fungi *Neurospora intermedia* has conventionally been used in an indigenous Indonesian food—oncom—and categorized as ‘edible’ fungi. *Neurospora intermedia* is among the fastest growing of all filamentous fungi and has shown considerable growth in solutions containing starch and xylan [9,10]. This fungus has been used for the production of ethanol [10,11], protein-rich biomass [9], and pigment [8,12]. Nevertheless, research using *N. intermedia* for enzyme production is limited in the literature. The primary purpose of this study was to evaluate the potential of *Neurospora intermedia* to produce amylase and xylanase in liquid fermentation. The study is further extended to the characterization of these enzymes to explore their suitability for application in industry. To the best of our knowledge, this is the first report on amylase and xylanase production from *N. intermedia* in submerged fermentation.

## 2. Results and Discussion

*N. intermedia* is an edible fungus which grows fast and produces a high amount of protein-rich biomass. Considering these features, several investigations have been performed on this fungus for various purposes. Valorization of numerous industrial waste streams, such as straw and bran (lignocellulosic waste) [13,14,15], whole and thin stillage (starch-to-ethanol process waste) [9,10,11,16], vinasse (sugar-to-ethanol process waste) [17], and cream, cheese-whey, yoghurt and milk (dairy waste products) [18,19], along with production of ethanol [10,11], protein-rich fungal biomass for feed applications [10,11], and pigments [8,12], are samples of *N. intermedia* applications in various fields. Despite the great potential of this fungus for industrial applications, particularly in the biorefinery, the enzyme production potential of this fungus has not yet been investigated thoroughly. However, simultaneous enzyme production by this fungus can improve the yield and reduce the cost of the biorefinery. In this study, for the first time, production of two useful biorefinery enzymes—amylase and xylanase—by *N. intermedia* in submerged fermentation were investigated. The results are presented as follows.

### 2.1. Optimization of Enzyme Production in a Shake-Flask System

The microbial production of enzymes is significantly influenced by the components of the culture medium, especially the carbon and nitrogen sources. In addition, the nitrogen source can influence the pH of the medium, which may affect the enzyme activity and stability [20]. In order to investigate the influence of a nitrogen source on *N. intermedia* enzyme production, amylase and xylanase were produced in shake flasks containing medium supplemented with different nitrogen sources. No intracellular enzyme activity was detected. However, in the literature, intracellular amylase and xylanase production, by *Aspergillus niger* [21] and *Neurospora crassa* [22], respectively, were observed.

**Figure 1:**
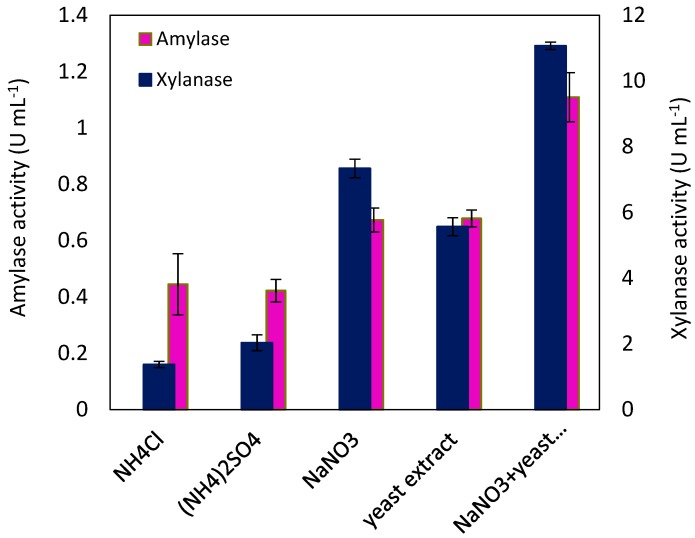
Effect of nitrogen sources on amylase and xylanase production by *Neurospora intermedia*, 10 g L^−1^ of single nitrogen sources, and 8 g L^−1^ NaNO_3_ and 2 g L^−1^ yeast extract in the combination of NaNO_3_ and yeast extract.

The maximum activity of extracellular enzymes in each medium was obtained after 48 h, as shown in Figure 1. An addition of NaNO_3_ to the basal medium resulted in the higher amylase and xylanase activity in comparison with the supplementation of the basal medium with NH_4_Cl or (NH_4_)_2_SO_4_. Higher enzyme activity observed in the medium containing NaNO_3_ may be rooted in two facts: either the amount of amylase and xylanase produced in this medium was higher than that in the media containing NH_4_Cl or (NH_4_)_2_SO_4_, or the enzymes produced in this medium had higher activity, which lies in the action of available ions as the enzyme cofactor.

While yeast extract resulted in excessive growth of *N. intermedia*, it also resulted in lower enzyme activity, especially xylanase, which can probably be attributed to the presence of simple sugars in the yeast extract. However, when 8 g L^−1^ NaNO_3_ and 2 g L^−1^ yeast extract were combined, the amylase and xylanase activity increased 2.52- and 8.07-fold compared to an addition of NH_4_Cl. Furthermore, a comparison between the variation of amylase and xylanase activity in the media containing different nitrogen sources revealed a higher sensitivity of xylanase toward the nitrogen source. Similarly, NaNO_3_ was identified as the best nitrogen source, among different single nitrogen sources, in the production of amylase by *N. crassa* [23]. NaNO_3_ has also been referred to in the literature as a good nitrogen source for xylanase production in different microorganisms, especially filamentous fungus [20].

### 2.2. Enzyme Production in a Bubble Column Reactor

In order to investigate the effect of agitation and aeration on *N. intermedia* enzyme production, the fungus was cultivated in the optimum medium, identified for each enzyme in the shake-flask system, and cultivation was conducted in a bubble column bioreactor. Due to the high growth of *N. intermedia* in the solution, resulting in high viscosity, agitation and aeration are required during fungal growth and enzyme production. In contrast with a stirred tank reactor, pneumatically agitated bioreactors, such as bubble column bioreactors, cause lower shear stress and better mass transfer [24]. *N. intermedia* showed an acceptable growth in both starch (5.07 ± 0.36 g dry biomass L^−1^) and xylan (7.74 ± 0.68 g dry biomass L^−1^) medium.

**Figure 2:**
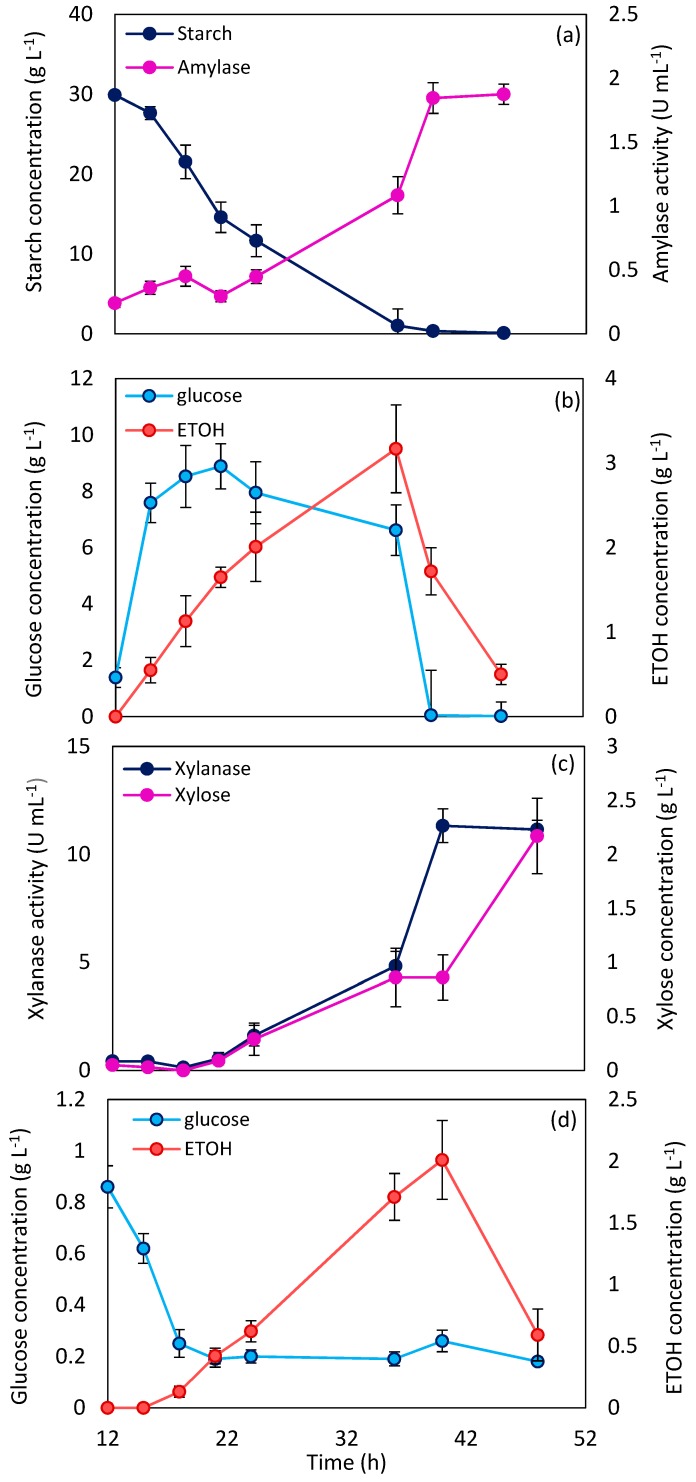
Starch and amylase concentrations (**a**), glucose and ethanol (ETOH) concentrations (**b**) in the starch medium, and xylanase production and xylose release (**c**), glucose and ethanol (ETOH) concentrations (**d**) in the xylan medium during cultivation of *N. intermedia* at 35 °C in the bubble column bioreactor.

Amylase production began after 12 h, which can be considered as the *N. intermedia* lag phase, as shown in Figure 2a. During the cultivation process, the starch content of the medium decreased by the increase in amylase activity, resulting in 98.8% starch consumption after 39 h of fermentation. However, an unexpected drop in amylase activity was observed around 20 h after cultivation, which can be attributed to a change in the pH of the culture medium. Starch hydrolysis by *N. intermedia* amylase led to the release of glucose, which was consumed by fungus, and resulted in up to 3.17 ± 0.52 g L^−1^ ethanol, as shown in Figure 2b.

A similar trend was observed for xylan and xylanase. Xylanase activity reached 11.32 ± 0.78 U mL^−1^ after 40 h of cultivation and did not show any significant changes after that. As a consequence of xylanase function, xylan was hydrolyzed and xylose was released (up to 2.17 ± 0.35 g L^−1^), as shown in Figure 2c. The trend of glucose changes during fermentation; Figure 2d shows consumption of the glucose inducer by *N. intermedia*, in the beginning of fermentation, after which it remains almost constant. The constant amount of glucose, along with an increasing trend of ethanol concentration, confirmed the consumption of xylose by *N. intermedia* at this stage. A similar report of xylose consumption by *N. intermedia* has been presented by Batori et al. [9].

In both starch and xylan media, after almost 40 h, ethanol concentration decreased in the course of time, which coincided with the stop in enzyme production. Ethanol consumption by *N. intermedia* and evaporation can be responsible for the reduction in ethanol concentration. A decrease in ethanol concentration during *N. intermedia* growth in different media has been reported previously [9,10,15].

Comparing the results of enzyme production in shake-flask and bubble column bioreactor systems showed that the aeration brought about a significant effect on the maximum enzyme production (39% and 2.3% increase in amylase and xylanase production, respectively). In addition, it accelerated the fermentation process and enzyme production (16.6% reduction in fermentation time). Proper aeration in the bubble column bioreactor can provide an appropriate gas–liquid interaction area followed by suitable mass transfer. Results of previous studies of *N. intermedia* cultivation in the airlift and bubble column bioreactor showed that this fungus has benefited from appropriate aeration [10]. An increase in enzyme production with a simultaneous decrease in fermentation time, as consequences of appropriate aeration, have been reported previously in the enzyme production by aerobic microorganisms [25].

### 2.3. Amylase and Xylanase Characteristics

#### 2.3.1. Effect of Temperature on Enzyme Activity

Enzyme activity is generally defined as the amount of a certain substrate converted per unit time, which is a function of active enzyme concentration and its specific reaction rate constant [26]. In accordance with Arrhenius law (Equation (1)), the reaction rate constant (k) increases with increasing temperature (T).

(1) $$k\;=\;A\;e^{\frac{-E_{a}}{RT}}$$

**Figure 3:**
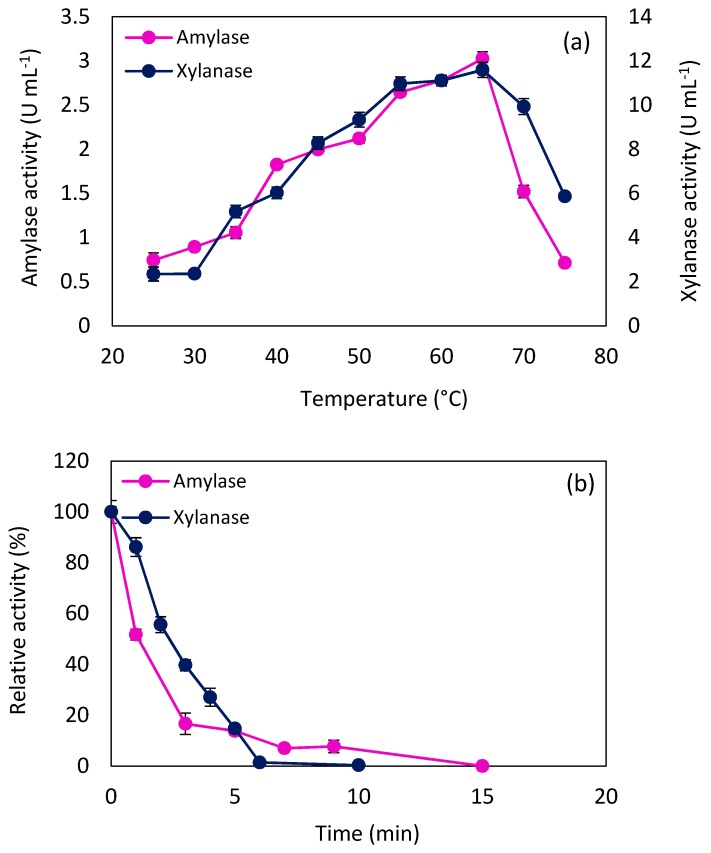
*N. intermedia* amylase and xylanase (**a**) activity in different temperatures, (**b**) stability at 65 °C.

Consequently, it would be expected to observe an increasing trend in enzyme activity along with increasing temperature. However, at high temperatures, enzymes start to deactivate and lose their activity partially or entirely. Under these circumstances, enzyme stability turns out to be important and considerable. The experimental results, as shown in Figure 3a, clearly show that both amylase and xylanase activity increased sharply until 65 °C, at which point the maximum activity of amylase (3.02 ± 0.08 U mL^−1^) and xylanase (11.59 ± 0.34 U mL^−1^) were reached. Beyond this point, a significant decline in both enzyme activities were observed. Wide ranges of optimum temperature have been reported for amylase and xylanase derived from various fungi and bacteria. Nevertheless, results of amylase and xylanase optimum temperature, in this study, are in agreement with earlier studies of the enzymes produced by *Aspergillus* [27,28].

The optimum reaction temperature data can be applied in the Arrhenius equation to calculate the activation energy required for each substrate hydrolysis. At the beginning of the reaction, the substrate concentration is high, and the rate of reaction is independent of the substrate concentration. Therefore, the Arrhenius equation can be written as Equation (2).

(2) $$\ln V_{max}\;=\;\frac{-E_{a}}{RT}$$

where, V_max_ is the enzyme activity; and E_a_ is the substrate hydrolysis activation energy (kJ mol^−1^). Considering the temperature-dependent binds between the enzymes and their polymeric substrates (starch and xylan), activation energies for hydrolyzing these substrates can be regarded as the apparent activation energy. Apparent activation energies (E_a, app_) for starch and xylan hydrolysis by *N. intermedia* enzymes, in different ranges of temperature, are reported in Table 1, which are comparable to the starch and xylan hydrolysis activation energy of *Rhizopus microsporus* amylase (34.3 kJ mol^−1^) [29] and *Fusarium sp.* xylanase (37.15 kJ mol^−1^) [30], respectively.

Different conformation of the enzyme would result in changes in the slope of the Arrhenius plot and the enzyme activation energy [26]. The results in Table 1 show that both amylase and xylanase have single conformation up to the optimum temperature (65 °C). Positive values of E_a_ in the range of 25 to 65 °C, revealed that catalysis reactions prevail over enzymatic deactivation below the optimum point. Furthermore, the higher values of xylanase E_a_ in comparison with amylase E_a_ may propose that the required energy for formation of the activated complex in starch hydrolysis is less than that in hydrolysis of xylan. Above the optimum point, both amylase and xylanase showed less activity towards the hydrolysis of their substrates, resulting from the enzyme’s significant denaturation above the optimum reaction temperature.

Inadequate enzyme stability can give rise to economic losses when the enzyme is to be utilized. Therefore, in terms of the bioprocess design and economy perspective, it is crucial to investigate the thermal deactivation and realize the stability of the enzyme under specific operating conditions. In this regard, *N. intermedia* amylase and xylanase’s thermal deactivation were investigated by measuring the residual activity of enzymes at their optimum temperature, 65 °C, as shown in Figure 3b. Data obtained from the experimental study were fitted to the equation of thermal deactivation of enzymes (Equation (3)) as follows:

(3) $$E\;=\;E_{0}\times e^{-k_{d}t}$$

where, t is time (min); E_0_ is the initial enzyme activity (U mL^−1^); E is the enzyme activity (U mL^−1^) at time t; and k_d_ is the deactivation rate constant (min^−1^). Half-life values of the enzymes were also calculated by replacing E with E_0_/2 in Equation (3), as shown in Equation (4).

(4) $$t_{\frac{1}{2}}\;=\;\frac{\ln\left(2\right)}{k_{d}}$$

The comparison between the deactivation rate constant and half-life of *N. intermedia* enzymes, as shown in (Table 2, showed that amylase was the most stable at 65 °C (lower deactivation rate constant) and required 2.42 min to lose half of its activity, while xylanase lost half of its activity after 2.04 min.

Generally, enzyme thermostability is an inherent feature, with variation based on the enzyme source. However, it has been observed that some microorganisms produce enzymes with different thermal stability regarding their special growth temperature and conditions [31]. There are several reports of amylase and xylanase thermostability investigation in the literature, which shows different half-life values of amylase [32,33] and xylanase [34,35] at 65 °C.

#### 2.3.2. Effect of pH on Enzyme Activity

**Figure 4:**
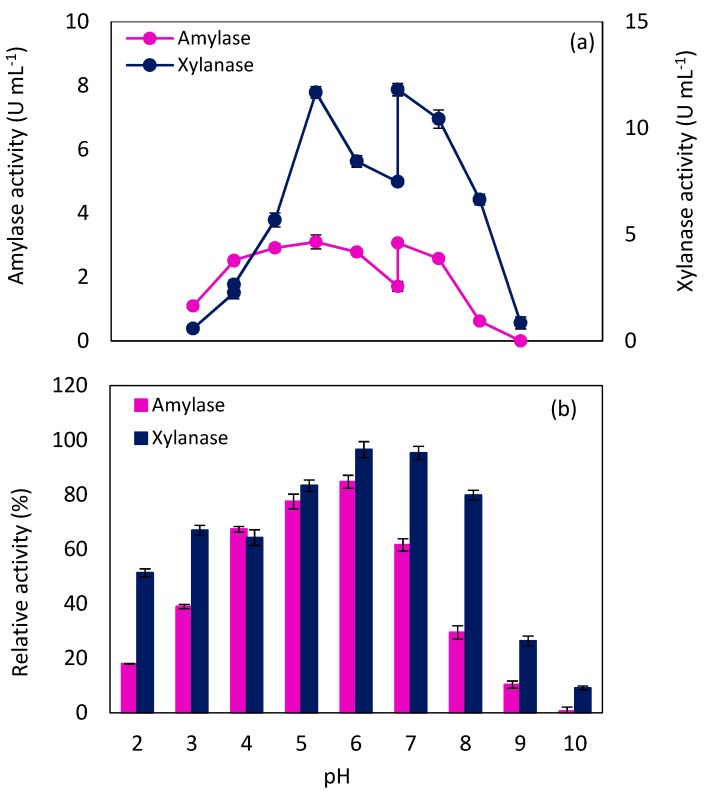
*N. intermedia* amylase and xylanase (**a**) activity and (**b**) stability in different pH at 65 °C after 4 h.

The other critical factor which affects the enzyme activity is pH. Each enzyme has its own optimum pH in which the active site can bind to the substrate tightly and catalyze the reaction favorably. In order to determine the optimum pH of amylase and xylanase produced by *N. intermedia*, their activity was examined in the pH range of 2.0 to 10.0, at 65 °C. Potential interaction by the buffer system was also investigated by measuring activity twice during buffer overlaps. The results shown in Figure 4a indicated that the maximum activity of amylase (3.10 ± 0.22 U mL^−1^) was obtained at pH 4.0–5.0, while xylanase activity reached the peak of (11.67 ± 0.28 U mL^−1^) at pH 5.0. As can be observed in Figure 4a, at pH 3.0, the change in buffer had no effect, whereas at pH 7.0, change in buffer type resulted in a dramatic transformation. This can probably be attributed to a presence of sodium in the sodium tetraborate buffer, which acted as a cofactor for both enzymes and enhanced the enzyme activity at pH 7.0. Similarly, optimum pH of amylase and xylanase in the pH range of 4–6 has been reported in previous studies [5,27].

The effect of pH on enzyme stability was also determined over the pH range of 2.0 to 10.0 for 4 h, as shown in Figure 4b. Xylanase retained more than 80% of its initial activity within a pH range of 5.0 to 8.0, while in the case of amylase, pH 6.0 created a situation in which more than 80% of initial activity was retained. For both enzymes, the activity decreased dramatically at pH values higher than 8.0.

#### 2.3.3. Kinetic Characteristics of Produced Enzymes

**Figure 5:**
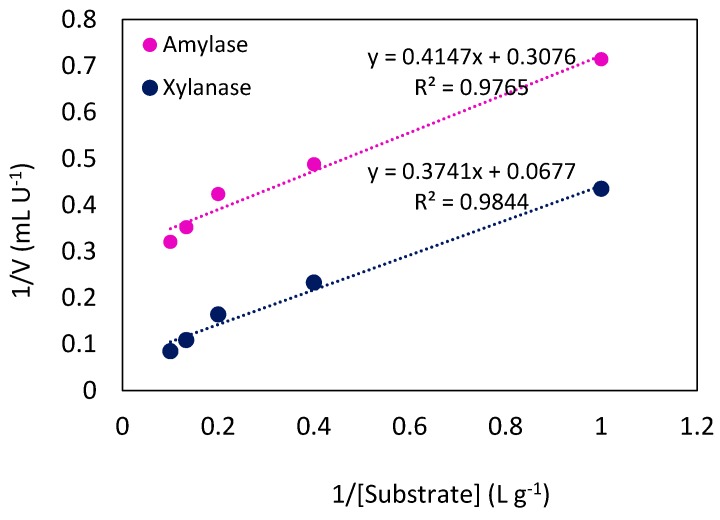
Lineweaver–Burk plot to calculate K_m, app_ and V_max, app_ of amylase and xylanase in hydrolysis of starch and xylan, respectively (under optimum conditions).

Kinetic studies on enzymes, by measuring the rate of catalyzed reaction under various conditions, can reveal the catalytic mechanism of the enzyme and the affinity with which the enzyme binds to its specific substrate. The kinetic of starch and beechwood xylan hydrolysis, using the Lineweaver–Burk plot, is depicted in Figure 5. Furthermore, the values of K_m, app_ and V_max, app_ are shown in Table 3.

The K_m_ value reflects the affinity of the enzyme towards its specific substrate, with smaller values representing greater affinity for its substrate [36]. Although it is not easy to compare the K_m_ and V_max_ values of different enzymes, as they depend on type of the substrate and conditions of the reaction, the values of K_m_ are in agreement with the previous reported range of K_m_ values for microbial xylanases (0.27–14 g L^−1^) [37] and amylases (0.19–29 g L^−1^) [6].

### 2.4. Enzyme Production by N. Intermedia through Wheat-Based Biorefinery

**Figure 6:**
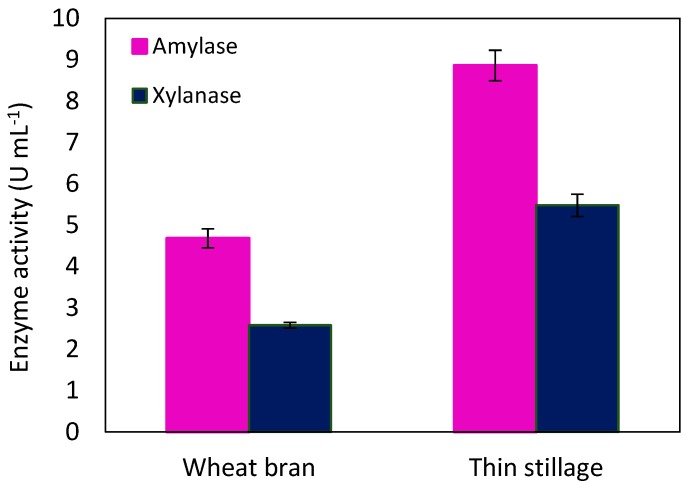
Enzyme production by *N. intermedia* cultivation in wheat bran and thin stillage media at 35 °C and pH = 5.5, after 48 h.

Restructuring the conventional fermentation industry into possible biorefineries for the production of enzymes brings considerable benefits, including reducing the cost and energy of the enzyme production, performing substrate hydrolysis during on-site enzyme production, and addressing the issue of waste stream disposal. Considering the tremendous consumption of wheat and its derivatives like wheat bran in the food and feed industry, wheat-based biorefineries have attracted much interest throughout the world. That is why this study was continued by cultivation of *N. intermedia* in thin stillage, a residual stream of the whole-wheat ethanol process, and a medium containing wheat bran, a byproduct of the wheat milling industry, to investigate enzyme production in a wheat-based biorefinery. The results in Figure 6 show that *N. intermedia* represented a significant potential in enzyme production from both media. However, lower starch (5.1 ± 0.048 g L^−1^) and xylan (4.68 ± 0.57 g L^−1^) content of wheat bran medium resulted in lower amylase (917.64 ± 45.09 U g starch^−1^) and xylanase (551.2 ± 14.95 U g xylan^−1^) production yield in this medium. Furthermore, the presence of pretreated wheat along with the relevant amount of mineral, carbon, and nitrogen sources in thin stillage can be the other reason for higher amylase (1004.53 ± 41.95 U g starch^−1^) and xylanase (1022.38 ± 50.37 U g xylan^−1^) production yield in thin stillage [10].

Amylase production from thin stillage has been also investigated by cultivation of *Aspergillus oryzae*, *Penicillium purpurogenum*, *Rhizopus*, *Mucor,* and *Monilia*. Maximum amylolytic activity was reported as 3.3 U mL^−1^ resulting from cultivation of *Aspergillus oryzae* [38], which was 62% less than activity observed from cultivation of *N. intermedia* in the current research.

Furthermore, in the literature there are other reports of integrated enzyme production by wheat-based biorefineries in solid or submerged fermentation. As an illustration, cultivation of *Aspergillus awamori* in a crude broth of wheat-flour resulted in the production of glucoamylase, protease, and phosphatase, and simultaneous hydrolysis of wheat starch, protein, and phytic acid content [39]. However, this is the first report of enzyme production through the wheat-based biorefinery by edible fungus *Neurospora intermedia*, which can be further beneficial in protein-rich biomass production for feed applications.

## 3. Materials and Methods

### 3.1. Microorganism

The edible fungus, originally from Indonesia, *Neurospora intermedia* CBS 131.92 (Centraalbureau voor Schimmelcultures, Utrecht, The Netherlands) was used to produce the enzymes in this study. The fungus was maintained on potato dextrose agar or PDA (composed of 20 g L^−1^ glucose, 4 g L^−1^ potato infusion, and 15 g L^−1^ agar) at 30 °C for 3–5 days followed by storage at 4 °C. The inoculum was prepared aseptically by adding 10 mL sterile distilled water to the sporulated plates and gently agitating the mycelia. The total spore count in the inoculum was determined by the Neubauer counting method. All liquid cultures were inoculated with 10 mL L^−1^ of spore solution containing 6.5 ± 0.4 × 10^5^ spores mL^−1^.

### 3.2. Substrate Media

Amylase and xylanase production by *N. intermedia* were investigated in synthetic and semisynthetic media and an industrial residue stream. Synthetic media were prepared using 30 g L^−1^ of starch or xylan, as a carbon source for amylase and xylanase production, respectively. This was supplemented with 1 g L^−1^ glucose (as an inducer), required nutrients (2.25 g L^−1^ MgSO_4_∙7H_2_O, 3.5 g L^−1^ KH_2_PO_4_, 1 g L^−1^ CaCl_2_∙2H_2_O, 10 mL L^−1^ trace metal containing 3 g L^−1^ EDTA, 0.9 g L^−1^ ZnSO_4_∙7H_2_O, 0.6 g L^−1^ FeSO_4_∙7H_2_O, 0.2 g L^−1^ H_3_BO_3_, 0.19 g L^−1^ MnCl_2_ 4H_2_O, 0.8 g L^−1^ Na_2_MoO_4_ 2H_2_O, 0.6 g L^−1^ CoCl_2_ 2H_2_O, 0.6 g L^−1^ CuSO_4_ 5H_2_O, 0.02 g L^−1^ KI) and 10 g L^−1^ of a nitrogen source, which is explained in Section 3.3. In the semisynthetic media, 30 g L^−1^ of wheat bran was used as a carbon source and the other components were the same as which were utilized in synthetic media. The thin stillage, from a bioethanol production process based mainly on wheat, was provided by Lantmännen Agroetanol (Norrköping, Sweden). Compositions of the wheat bran and thin stillage are shown in Table 4. All media were adjusted to pH 5.5 with 10 M NaOH and 6 M HCl.

### 3.3. Enzyme Production in Shake Flasks and Nitrogen Source

Amylase and xylanase production were investigated in 250 mL shake flasks. The flasks containing 100 mL of cultivation medium were sterilized (at 121 °C for 20 min), and after cooling, inoculated with the spore suspension followed by incubation at 35 °C on a rotary shaker (150 rpm). From incubated cultures sampling was carried out at regular time intervals. The culture broths were clarified by centrifugation at 8000× *g* for 15 min and the supernatant was used as a crude enzyme extract to determine the activity of enzymes.

In order to investigate the effect of a nitrogen source on enzyme production, the basal synthetic medium of each enzyme was supplemented with 10 g L^−1^ of different nitrogen sources (NH_4_Cl, (NH_4_)_2_SO_4_, NaNO_3_, yeast extract, and a combination of NaNO_3_ and yeast extract), in separated runs. Submerge fermentation was carried out as explained in Section 3.3.

### 3.4. Enzyme Production in a Bubble Column Bioreactor

Fungal cultivations were performed in 4.5 L bench bubble column bioreactors (Belach Bioteknik, Stockholm, Sweden). The bubble column bioreactor was filled with 3 L of the optimum medium for each enzyme, described in Section 3.2 and Section 3.3, in separate runs. The bioreactor containing medium was sterilized (at 120 °C for 3 h), and after cooling, spore suspension and antifoam were added to the cultivation medium. The solution pH was maintained at 5.5 (by 2 M NaOH), temperature was set at 35 °C and aeration, at a rate of 1 vvm (volume of air per volume of reactor per min), was fulfilled by passing the inlet air through a 0.2 μm pore size sterile polytetrafluoroethylene filter followed by a 90 μm pore size stainless steel sparger. Cultivation was performed for 48 h and samples were taken periodically, centrifuged, and analyzed. At the end of each cultivation, the fungal biomass was removed from the cultivation medium by filtration through a metal sieve. The mycelia were washed with distilled water, dried, and measured gravimetrically and the medium, as an enzyme crude extract, was stored at 4 °C for further analysis.

### 3.5. Enzyme Characteristics

#### 3.5.1. Effect of pH and Temperature on Enzyme Activity

The optimum pH of amylase and xylanase was investigated in a pH range of 2.0 to 10.0. To evaluate the pH stability, enzymes were preserved in different buffers at room temperature for 4 h and the remaining enzyme activity was measured. The applied buffer systems were citric acid buffer for pH 2.0–3.0, phosphate-citrate buffer for pH 3.0–7.0, and sodium tetraborate buffer for pH 7.0–10.0. To investigate the influence of temperature and determine the best temperature for enzyme operation, amylase and xylanase activity, separately, were tested at different temperatures (25–75 °C). Thermal stability was investigated by determination of enzyme activity after different pre-incubation times (0–30 min) at the optimum temperature of enzyme operation.

#### 3.5.2. Kinetic Properties of the Enzyme-Substrate System

The effect of substrate concentration on enzyme–substrate reaction kinetics was investigated using starch and beechwood xylan (1 to 10 g L^−1^) under optimum assay conditions of amylase and xylanase, respectively. Apparent Michaelis-constant (K_m, app_) and maximum velocity (V_max, app_) values of the enzymes were calculated from the slope and intercept of the straight-line plot of V^−1^ vs. S^−1^ (Lineweaver–Burk plot) as follows:

(5) $$\frac{1}{V}\;=\;\frac{K_{m}}{V_{max}}\frac{1}{\left[S\right]}+\frac{1}{V_{max}}$$

### 3.6. Analytical Methods

The amylolytic and xylanolytic activity of the *N. intermedia* crude enzyme extract was determined spectrophotometrically based on the measurement of the released reducing sugar by the dinitrosalicylic acid reagent [41]. An amylolytic activity assay was carried out based on the hydrolysis of 1% starch (*w*/*v*) at 40 °C in 0.2 M sodium acetate buffer (pH 4.8). Xylanolytic activity was measured by the hydrolysis of 1% beechwood xylan (*w*/*v*) at 50 °C in 0.05 M sodium citrate buffer (pH 5.3) [42]. One unit of enzyme activity was defined as the amount of enzyme required to liberate one μmole of reduced sugar, maltose and xylose in the case of amylase and xylanase, respectively, per min under assay conditions. The enzyme assay conditions were as stated above except where otherwise specified.

Investigation of the intracellular enzyme production was carried out using two methods of cell disruption [43]. At the end of fermentation, *N. intermedia* mycelia (10% *w*/*v*) were suspended in 10 mL of cold citrate buffer (pH 5 at 4 °C) and placed in a salt-iced water bath ultrasonic homogenizer (2510 Branson from Branson Ultrasonics Corporation, Danbury, CT, USA) at 42 kHz. In order to prevent enzyme thermal denaturation, sonication was carried out in 30 s intervals for a total time of 10 min. As the second method, mycelia were suspended in 0.2% (*v*/*v*) aqueous solutions of Triton X-100 to give a working concentration of 10% cell (*w*/*v*) with shaking (200 rpm) for three different durations (30, 60, and 120 min). The obtained suspensions from each method were centrifuged at 14,000× *g* at 4 °C for 10 min and the subsequent supernatant was examined for enzyme activity.

Starch contents of solutions, in the case of amylase production, were measured based on the color development that resulted from iodine binding to the starch polymers [44]. Glucose, ethanol, and total sugar were analyzed using a high performance liquid chromatography (HPLC) system (Waters 2695, Waters Corporation, Milford, MA, USA) [10].

## 4. Conclusions

The results confirm that *N. intermedia* possess the great ability in extracellular amylase and xylanase production. At the optimum temperature of hydrolysis reactions, 65 °C, *N. intermedia* amylase represented more stability and greater substrate affinity. However, its maximum substrate hydrolyzing rate was less than xylanase. Both enzymes produced by *N. intermedia* showed their optimum activity in pH around 5 and retained more than 50% of their initial activity in the pH range of 4 to 7 after 4 h. Relatively high enzyme production through cultivation of *N. intermedia* in thin stillage and the medium of wheat bran makes this fungus an appropriate candidate in enzyme production through a wheat-based biorefinery.

## Figures and Tables

**Table 1:** Apparent activation energies for starch and xylan hydrolysis by *N. intermedia* amylase and xylanase, respectively.

Temperature Range (°C)	E_a, app_ (kJ mol^−1^)	
	Amylase	Xylanase
25–65	30.97	36.65
65–75	−141.570	−66.52

**Table 2:** Deactivation rate constant and half-life of *N. intermedia* enzymes at 65 °C.

Enzyme	k_d_ (min^−1^)	$t_{\frac{1}{2}}$ (min)
Amylase	0.2862	2.42
Xylanase	0.3390	2.04

**Table 3:** Apparent kinetic constants for hydrolysis of starch and beechwood xylan by *N. intermedia* amylase and xylanase, respectively.

Enzyme	K_m, app_ (g L^−1^)	V_max, app_ (U mL^−1^)
Amylase	1.35	3.25
Xylanase	5.52	14.77

**Table 4:** Compositions of the wheat bran and thin stillage used in this study.

Component	g g^−1^ Wheat Bran *(dry basis)* [40]	g L^−1^ Thin Stillage [10]
*Solid fraction*		
Arabinan	0.088 ± 0.001	0.242 ± 0.140
Crude protein	0.147 ± 0.001	27.5 ± 1.25
Galactan	<0.001	0.175 ± 0.0420
Glucan	0.204 ± 0.02	3.182 ± 0.383
Mannan	<0.001	0.488 ± 0.234
Starch	0.17 ± 0.0016	<0.001
Xylan	0.156 ± 0.019	0.907 ± 0.419
*Dissolved saccharides*		
Arabinos		4.4 ± 0.2
Galactose		1.6 ± 0.2
Glucose		9.8 ± 0.7
Mannose		1.4 ± 0.2
Xylose		6.1 ± 0.4
Starch (using glucose as an indicator)		8.82 ± 0.63
Xylan (using xylose as an indicator)		5.36 ± 0.35
